# Cardiovascular Effects of *Trans*-4-Methoxy-β-Nitrostyrene in Spontaneously Hypertensive Rats: Comparison With Its Parent Drug β-Nitrostyrene

**DOI:** 10.3389/fphar.2019.01407

**Published:** 2019-11-29

**Authors:** Thayane Rebeca Alves-Santos, Odair Alves Silva, Hicla Stefany Moreira, Rosivaldo Santos Borges, Gloria Pinto Duarte, Pedro Jorges Caldas Magalhães, Saad Lahlou

**Affiliations:** ^1^Department of Physiology and Pharmacology, Federal University of Pernambuco, Recife, Brazil; ^2^Department of Pharmacy, Federal University of Pará, Belém, Brazil; ^3^Laboratory of Cardiovascular Pharmacology, Department of Physiology, Pharmacology, School of Medicine, Federal University of Ceará, Fortaleza, Brazil

**Keywords:** perineural capsaicin pretreatment, sensory C-fibers, *trans*-4-methoxy-β-nitrostyrene, vago-vagal reflex, β-nitrostyrene

## Abstract

We previously reported that *trans*-4-methoxy-β-nitrostyrene (T4MN) evoked higher vasorelaxant effects in small resistance arteries from spontaneously hypertensive rats (SHRs) in comparison with its parent drug, the β-nitrostyrene 1-nitro-2-phenylethene (NPe). To further our knowledge of the influence of insertion of an electron-releasing group such as methoxy in the aromatic ring of NPe, we investigated the cardiovascular responses to intravenous (i.v.) injection of T4MN in SHRs and compared with those of NPe. In anesthetized SHRs, i.v. treatment with T4MN (0.03–0.5 mg/kg) and NPe (0.03–3 mg/kg) induced dose-dependent bradycardia and hypotension, which were biphasic (named phases 1 and 2). Magnitude of these responses was significantly higher for T4MN compared with NPe. Phase 1 cardiovascular responses to both T4MN (0.3 mg/kg) and NPe (3 mg/kg) were prevented by cervical bivagotomy or perineural treatment of both cervical vagus nerves with capsaicin, but was unchanged by i.v. pretreatment with capsazepine or ondansetron. After injection into the left ventricle, NPe and T4MN no longer evoked phase 1 responses. In conscious SHRs, NPe (3 mg/kg, i.v.), and T4MN (0.3 mg/kg, i.v.) evoked monophasic hypotensive and bradycardiac effects which were suppressed by i.v. pretreatment with methylatropine. It is concluded that i.v. administration of NPe and T4MN in SHRs induced a vago-vagal hypotensive and bradycardic reflex that did not involve the activation of vanilloid TRPV_1_ or 5-HT_3_ receptors located on vagal pulmonary sensory nerves. With respect to its parent drug, T4MN was more potent in inducing this reflex. Phase 2 hypotensive response to i.v. NPe and T4MN seems partially resulting from a direct vasodilatory action. It seems that insertion of a methoxy group into the aromatic ring stabilized NPe, which in turn increases its cardiovascular effects.

## Introduction

Previously, we showed that 1-nitro-2-phenylethane (NPa), the unique nitro compound isolated from plants (Gottlieb and Magalhães, 1960), evoked interesting vasorelaxant effects in isolated aortic and small resistance artery preparations from both normotensive and spontaneously hypertensive rats (SHRs) (de Siqueira et al., 2010; Interaminense et al., 2013; Brito et al., 2013). NPa-induced vasorelaxation involves the activation of the soluble guanylate cyclase (sGC)/cyclic guanosine monophosphate (cGMP) in a nitric oxide (NO)-*independent* manner, resulting in increased intracellular cGMP levels (Brito et al., 2013). Conformational restriction of the molecule of NPa resulted in the formation of 1-nitro-2-phenylethene (NPe), a β-nitrostyrene derivative. In rat aortic preparations, we showed that NPe was about 3.5 times more potent as a vasorelaxing agent than its parent drug, NPa (Arruda-Barbosa et al., 2014).

In order to assess the influence of electronic structural modifications, we synthesized several new nitroderivatives in which electron donors were bonded in the aromatic ring of NPe. Previous *in vitro* experiments using aortic rings from normotensive rats showed that the methoxy derivative, *trans*-4-methoxy-β-nitrostyrene (T4MN), displayed vasorelaxing activity through a similar mechanism as described for NPa (Arruda-Barbosa et al., 2017). In contrast, using resistance mesenteric artery preparations from SHRs, Alves-Santos and coworkers showed that T4MN evoked a potent vasorelaxation that was mediated through both endothelium-dependent (activation of Akt/eNOS/NO pathway) and endothelium-independent (activation of sGC/cGMP/PKG pathway) mechanisms (Alves-Santos et al., 2019). Interestingly, the potency of vasorelaxation of T4MN in resistance arteries was 22-fold greater than that of its parent drug, NPe (Alves-Santos et al., 2019), an effect that seems indicative that insertion of an electron donor such as a methoxy group into the aromatic ring stabilized the NPe molecule, which in turn increases its vasorelaxant potency.

The present study was undertaken to investigate whether such an enhancement occurs for the cardiovascular responses of intravenous (i.v.) injection of T4MN in SHRs. Indeed, the putative mechanism of these responses was also studied. Previous *in vivo* studies performed in normotensive rats showed that intravenously injected NPa and *trans*-4-methyl-β-nitrostyrene evoked a vago-vagal bradycardiac and hypotensive responses that occurred as rapidly as the vago-vagal reflex responses elicited by i.v. capsaicin (Donnerer and Lembeck, 1982; Yang et al., 1993) or serotonin (Owen et al., 2005). The vago-vagal reflex evoked by NPa did not involve activation of either vanilloid TRPV_1_ or 5-HT_3_ receptors (de Siqueira et al., 2010), while that elicited by *trans*-4-methyl-β-nitrostyrene activated vanilloid TRPV_1_, but not purinergic (P2X) or 5-HT_3_ receptors located on vagal pulmonary sensory nerves (Teófilo et al., 2019).

## Materials and Methods

### Synthesis of NPe and T4MN

**Figure 1:**
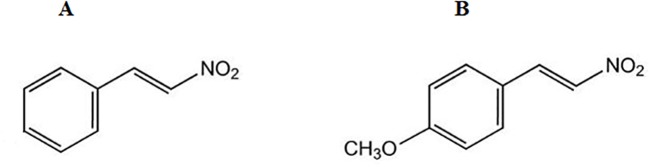
Chemical structure of 1-nitro-2-phenylethene **(A)** and *trans*-4-methoxy-β-nitrostyrene **(B)**.

The synthesis of the two nitroderivatives was performed at the Department of Pharmacy, Federal University of Pará, Belém, PA, Brazil. NPe or 1-((*E*)-2-nitro-vinyl)-benzene (β-nitrostyrene) (**Figure 1A**) as well as its methoxy derivative 1-((*E*)-2-nitro-vinyl)-(4-methoxy)-benzene or T4MN (**Figure 1B**) were synthesized by employing the Claisen–Schmitd’s procedure (Vogel, 1989; Ford et al., 1994) according to a previously described method (Arruda-Barbosa et al., 2014; Arruda-Barbosa et al., 2017, respectively).

### Solutions and Drugs

Sodium pentobarbital (Sanofi, Libourne, France) and heparin (Laboratoires Léo SA, Montigny-le-Bretonneux, France) were used as commercially available injectable solutions. Methylatropine bromide (a peripheral muscarinic antagonist), ondansetron hydrochloride (a 5-HT_3_ receptor antagonist), and capsaicin (a TRPV_1_ receptor agonist) were purchased from Sigma Chemical Co. (St. Louis, MO, USA), while capsazepine (a selective TRPV_1_ receptor antagonist) was purchased from Tocris (Ballwin, MO, USA). Penicillin G benzathine salt was purchased from Lafepe (Recife, PE, Brazil). All drugs were dissolved in saline solution, except for capsaicin which was dissolved in saline containing 1% Tween 80 and 1% ethanol. Likewise, capsazepine was first diluted in dimethyl sulfoxide to 0.1 mol/l (∼37 mg/ml) and further diluted with saline containing 10% Tween 80 and 10% ethanol, as previously described (Teófilo et al., 2019). NPe and T4MN were first dissolved in ethanol, and the desired concentration was achieved using sterile isotonic saline. Our previous investigations showed that this vehicle (0.1 ml) has no significant effects on either baseline mean arterial pressure (MAP) or heart rate (HR) in both normotensive and SHRs (de Siqueira et al., 2010; Interaminense et al., 2011; Teófilo et al., 2019). The volume of each bolus injection of the nitroderivatives was 0.1 ml. The venous catheter was then flushed by 0.2 ml (dead space). Methylatropine, capsazepine, and ondansetron were administered in a volume of 1 ml/kg body weight. Doses of drugs used herein were chosen according to those recommended in the literature. Systemic injections of nitroderivatives were separated by 10-min intervals in order to avoid tachyphylaxis.

### Animals

Adult male SHRs (age, 16–18 weeks) were obtained from our breeding facilities and were kept under conditions of constant temperature (22 ± 2°C) with a 12-h light/12-h dark cycle. SHRs were fed normal chow with diet and tap water provided *ad libitum*. All animals were handled according to the Guide for the Care and Use of Laboratory Animals of the Brazilian National Council for Animal Experimentation. All animal experiments were reviewed and approved by the institutional animal ethics committee, the “Comissão de Ética no Uso de Animais” (CEUA), Federal University of Ceará (23076.050472/2012-77).

### Catheterization Procedure

Under pentobarbital anesthesia (50 mg/kg, i.p.), the left femoral artery was catheterized with a polyethylene catheter (PE-10 fused PE-50) that was advanced until the tip will be positioned at the abdominal aorta just below the renal artery. This arterial catheter allows us to record the arterial blood pressure. For systemic drug administration, the inferior vena cava was cannulated through the left femoral vein as previously described (Alves-Santos et al., 2016). After surgical procedure, all animals received an intramuscular injection of penicillin (24,000 IU), then housed individually in plastic cages and allowed to recover for 24 h before any circulatory experiments.

### Experimental Design and Protocols

At the day of experiment, rats were initially anesthetized with sodium pentobarbital (50 mg/kg, i.p.). When necessary, supplement doses of pentobarbital were injected to maintain abolition of pain reflex evoked by pinching of the skin of the hindlimbs. Body temperature was maintained at 37°C by means of a controlled table placed under the animal lying under supine position. A short tracheal cannula was inserted after a tracheotomy, through which rats breathed spontaneously. In a set of animal (*n* = 5), a Teflon catheter connected to a blood pressure transducer was inserted into the right carotid artery and advanced until its tip was positioned in the left ventricle. The correct position of the intraventricular catheter was confirmed by measurement of left ventricular blood pressure and postmortem examination.

Thereafter, the arterial catheter was connected to a blood pressure transducer and baseline cardiovascular parameters were measured using an acquisition system (PowerLab 4/30, ML866; ADInstruments), as previously reported (Teófilo et al., 2019). After obtaining a stable MAP and HR tracing, six series of experiments were performed in anesthetized rats while series 7 was conducted in conscious SHRs.

#### Series 1

This series of experiments was carried out to establish a dose–effect relationship. Therefore, the time course of the changes in MAP and HR was recorded in each animal after i.v. injection of increasing bolus doses of NPe (0.03, 0.05, 0.1, 0.3, 0.5, 1, and 3 mg/kg) or T4MN (0.03, 0.05, 0.1, 0.3, and 0.5 mg/kg). Injection response times were measured from the end of an injection to the onset of bradycardia.

#### Series 2

It is well known that the vagus nerve is a mixed nerve which contains more afferent than efferent nerve fibers (Mei et al., 1980; Prechtl and Powley, 1990). To assess the role of these nerves fibers, maximal changes in MAP and HR elicited by NPe (3 mg/kg, i.v.) or T4MN (0.3 mg/kg, i.v.) were determined before and 10 min after cervical bivagotomy.

#### Series 3

In order to assess the role of vagal C-fiber afferents in mediation of the cardiovascular responses of T4MN and NPe, we performed a perineural treatment (PNT) with both cervical vagi, a well-known procedure to block selectively the neural conduction of vagal afferents (Jancsó and Such, 1983), as previously described (de Siqueira et al., 2010). Briefly, the right and left vagus nerves at the cervical level were isolated. Cotton swabs soaked with capsaicin (250 µg/ml) were placed around the nerves for 20 min. After removal of the swabs, the area was rinsed with saline. Maximal changes in MAP and HR evoked by NPe and T4MN (3 and 0.3 mg/kg, respectively) were determined before and after PNT with capsaicin.

#### Series 4

This series of experiments examined the putative involvement of vanilloid TRPV_1_ receptor activation in the mediation of cardiovascular responses to the two nitroderivatives. Hence, the maximal MAP and HR changes elicited by i.v.-administered NPe and T4MN (3 and 0.3 mg/kg, respectively) were determined in each animal before and 40 s after the i.v. pretreatment with the competitive TRPV_1_ receptor antagonist capsazepine (1 mg/kg, i.v.) (Malinowska et al., 2001).

#### Series 5

This series of experiments was performed to determine the putative involvement of serotoninergic (5-HT_3_) receptor activation in the mediation of cardiovascular responses to the two nitroderivatives. Therefore, the maximal MAP and HR changes elicited by i.v.-administered NPe and T4MN (3 and 0.3 mg/kg, respectively) were determined in each animal before and 5 min after the i.v. pretreatment with the 5-HT_3_ receptor antagonist ondansetron (30 µg/kg, i.v.) (Bagchi and Deshpande, 1999).

#### Series 6

To assess the location of the afferent C-fiber endings involved in elicitation of the observed cardiovascular responses, NPe and T4MN (3 and 0.3 mg/kg, respectively) were injected directly into the left ventricle. Such an injection bypasses the pulmonary circulation without immediate access to the pulmonary C-fibers. Responses to left ventricle injection were compared to those elicited by systemic injections of NPe and T4MN (3 and 0.3 mg/kg, respectively) performed 10 min earlier.

#### Series 7

In order to assess the role of cholinergic mechanism in the mediation of the observed cardiovascular changes, maximal changes in MAP and HR elicited by i.v. injection of NPe or T4MN (3 and 0.3 mg/kg, respectively) were determined in conscious SHRs that had been pretreated intravenously 10 min earlier with vehicle (1 ml/kg) or 1 mg/kg methylatropine (de Siqueira et al., 2006; de Siqueira et al., 2010).

### Statistical Analysis

Data are presented as mean ± SEM. Maximal changes in MAP and HR (expressed as a percentage of baseline values) after each dose of NPe or T4MN were used to construct a dose–response curve. MAP was calculated as diastolic arterial pressure + (systolic arterial pressure − diastolic arterial pressure)/3. The results were analyzed using paired Student’s *t* test, Mann–Whitney *U* test, and one- or two-way analysis of variance (ANOVA), followed by Dunnett’s multiple comparison tests when appropriate. A value of *P* < 0.05 was considered statistically significant.

## Results

In pentobarbital-anesthetized SHRs (*n* = 32) without any treatment, the average baseline MAP and HR values were 143.2 ± 6.7 mmHg and 310 ± 12 bpm, respectively.

### Cardiovascular Responses to Intravenous Injections of NPe and T4MN: Effects of Bilateral Vagotomy, Perineural Treatment With Capsaicin, and Pretreatment With Capsazepine or Ondansetron (Series 1 to 5)

**Figure 2:**
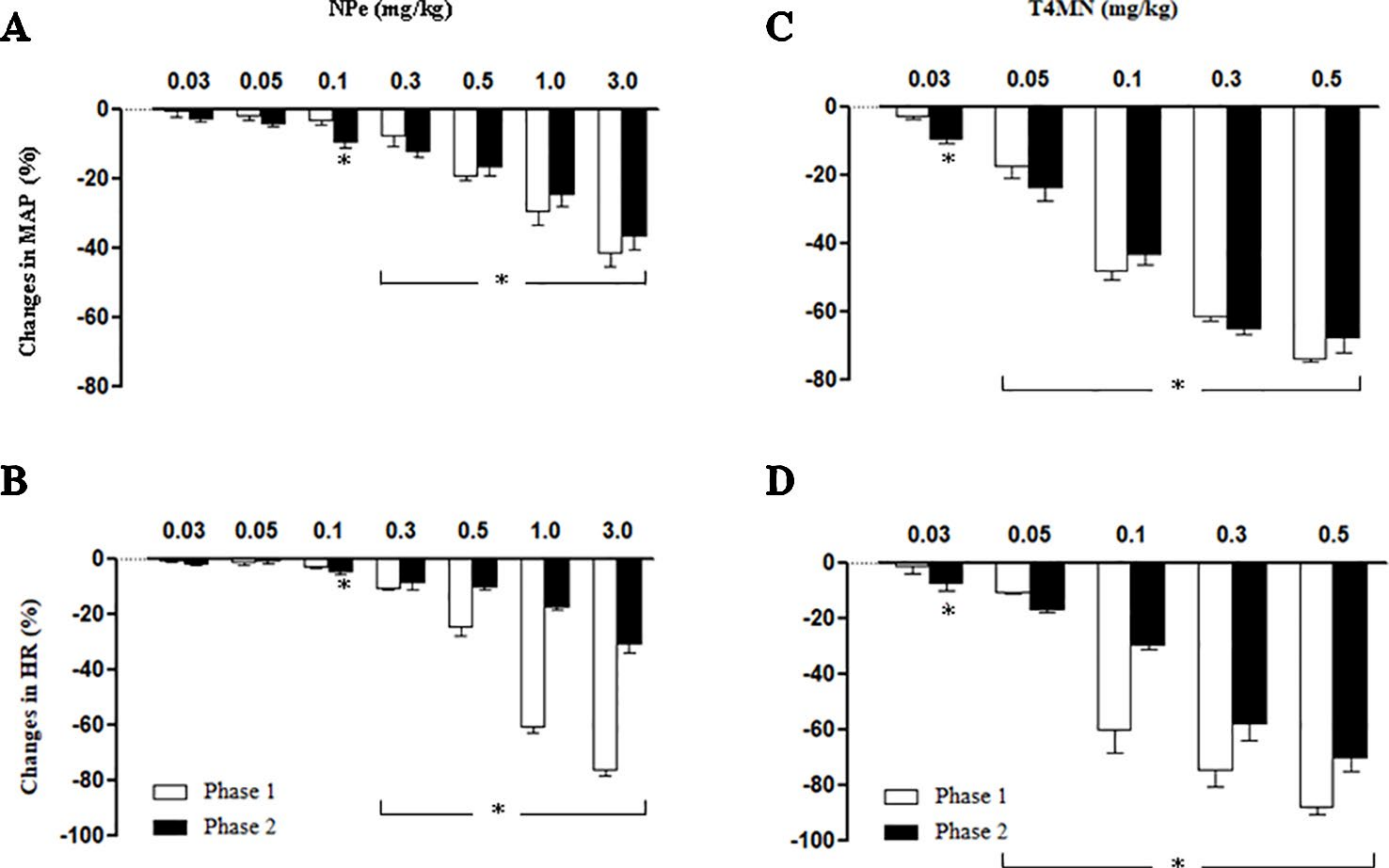
Maximal decreases in mean arterial pressure (MAP) and heart rate (HR) induced by intravenous injection of 1-nitro-2-phenylethene (NPe; 0.03–0.3 mg/kg) (**A** and **B**, respectively) and *trans*–4–methoxy–β–nitrostyrene (T4MN; 0.03–0.5 mg/kg) (**C** and **D**, respectively) in pentobarbital-anesthetized spontaneously hypertensive rats. Biphasic (phases 1 and 2) hypotensive and bradycardiac effects were evident at doses of ≥0.3 mg/kg for NPe and at doses of ≥0.05 mg/kg for T4MN. Note that phase 1 and phase 2 cardiovascular responses to both NPe and T4MN were dose-dependent (*P* < 0.01, one-way ANOVA). Data are the mean ± SEM and expressed as a percentage of baseline (*n* = 8 per group). *P < 0.05 by paired Student’s *t* test vs. the corresponding baseline values.

**Figure 3:**
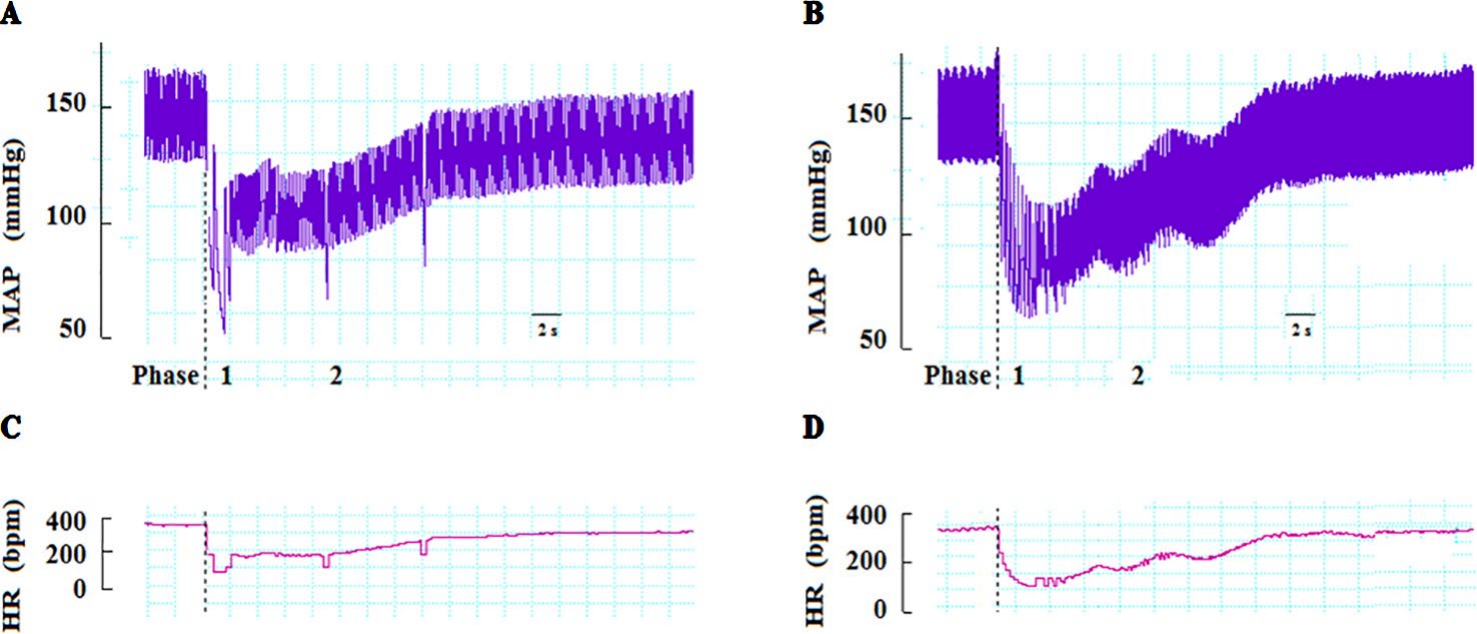
Representative recordings showing changes in mean arterial pressure (MAP) and heart rate (HR) induced by intravenous injection of NPe (3 mg/kg) (**A** and **C**, respectively) and T4MN (0.3 mg/kg) (**B** and **D**, respectively) in pentobarbital-anesthetized spontaneously hypertensive rats without any pretreatment. *Dotted lines* indicate the time of injection. *bpm* beats per minute.

In anesthetized rats, i.v. treatment with increasing doses of NPe (0.03–3 mg/kg) and T4MN (0.03–0.5 mg/kg) evoked dose‐dependent (*P* < 0.01, one‐way ANOVA) hypotensive (**Figures 2A, C**, respectively), and bradycardiac (**Figures 2B, D**, respectively) effects, which became significant (*P* < 0.05, paired Student’s test; **Figure 2**) at doses of 0.1 and 0.03 mg/kg, respectively. For doses from 0.3 to 3 mg/kg of NPe, the hypotensive (**Figure 2A**) and bradycardiac (**Figure 2C**) responses to NPe are significant and biphasic (characterized by phases 1 and 2). The same was observed for those elicited by doses from 0.05 to 0.5 mg/kg of T4MN (**Figures 2B, D**). The time latency of phase 1 component of NPe‐ and T4MN‐induced decreases in HR and MAP was 1–2 s after injection, while that of phase 2 component of bradycardia and hypotension occurred at 7–11 and 7–9 s after injection, respectively, for NPe and T4MN (**Figure 3**). Cardiovascular responses to NPe and T4MN both returned to baseline within 40–70 s. Two-way ANOVA revealed that both phase 1 and phase 2 hypotensive and bradycardiac effects elicited by T4MN (0.03–0.5 mg/kg, i.v.) were as significantly enhanced as those evoked by the same dose range of NPe (*P* < 0.001; **Figure 2**).

Cervical bivagotomy induced a significant increase in baseline HR (*P* < 0.05; **Table 1**) without altering baseline MAP. However, both baseline MAP and HR values were unaltered by PNT with capsaicin (250 µg/ml) (**Table 1**). The same was observed following pretreatment with capsazepine (1 mg/kg, i.v.) or ondansetron (30 µg/kg, i.v.) (**Table 1**). When the same dose of NPe (3 mg/kg, i.v.) or T4MN (0.3 mg/kg, i.v.) was re-injected in the same animal at 10-min interval, no tachyphylaxia phenomenon was observed for both phases 1 and 2 (data not shown).

**Table 1:** Effects of various pretreatments on basal mean arterial pressure (MAP) and heart rate (HR) in both pentobarbital-anesthetized and conscious, freely moving spontaneously hypertensive rats (SHRs) in which the cardiovascular effects of 1-nitro-2-phenylethene and *trans*–4–methoxy–β–nitrostyrene were investigated.

Pretreatment	*n*	MAP (mmHg)		HR (bpm)	
		Basal	After T	Basal	After T
**Anesthetized SHRs**					
Bivagotomy	6	146.0 ± 4.1	151.3 ± 0.8	315 ± 12	402 ± 6*
PNT	6	142.2 ± 2.6	137.4 ± 1.7	311 ± 9	316 ± 8
Capsazepine	6	139.7 ± 4.1	130.2 ± 5.4	300 ± 16	303 ± 12
Ondansetron	6	145.9 ± 6.3	154.1 ± 2.7	309 ± 13	303 ± 13
Intraventricular					
LV	6	142.1 ± 7.3	————–	304 ± 11	———
**Conscious SHRs**					
Methylatropine	5	170.5 ± 6	178.6 ± 8.7	321 ± 12	403 ± 12*

Data are the mean ± SEM. T treatment; PNT, perineural treatment; LV, left ventricle. *P < 0.05 by paired Student’s t test.

**Figure 4:**
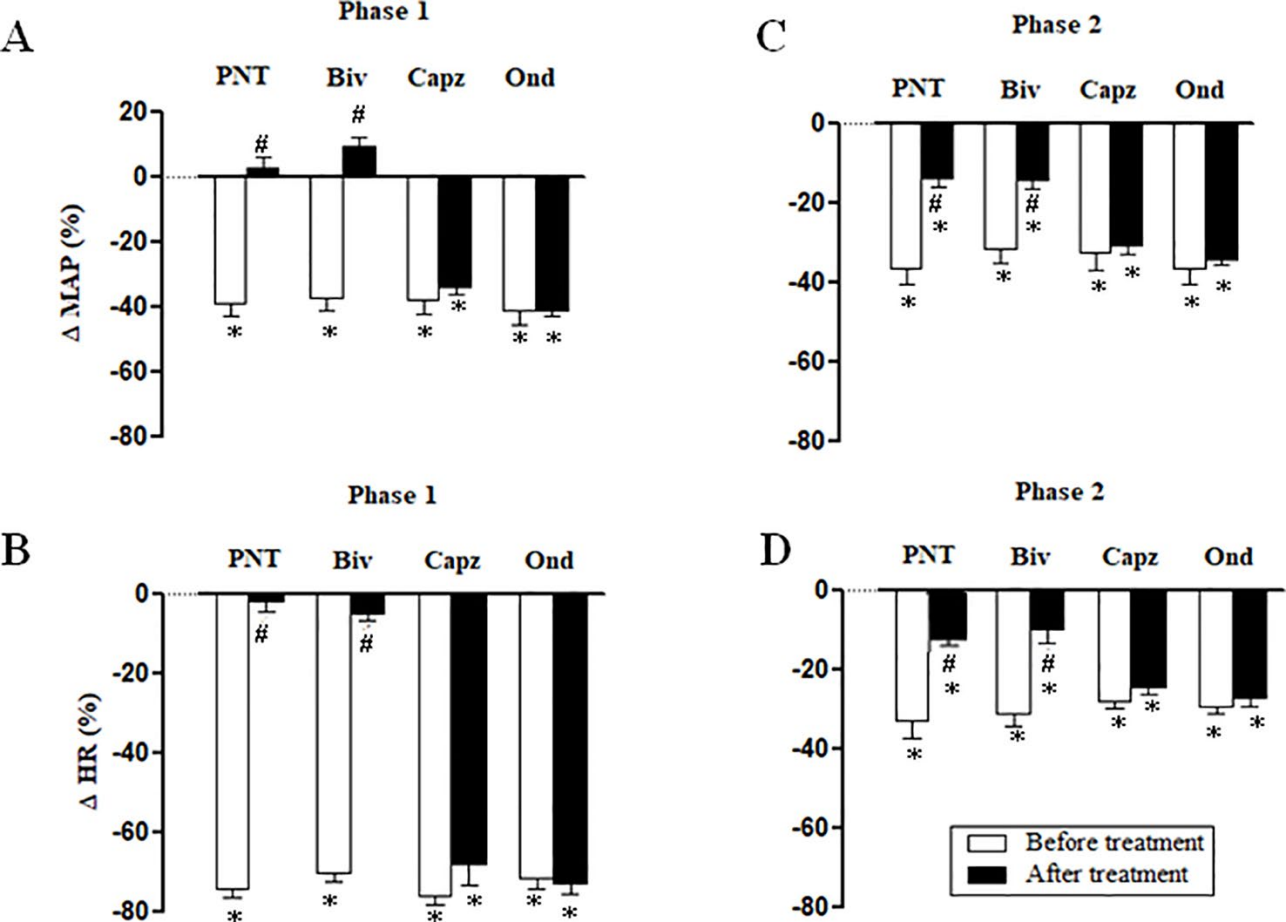
Phase 1 decreases in mean arterial pressure (ΔMAP) and heart rate (ΔHR) (**A** and **B**, respectively) and phase 2 hypotensive and bradycardiac effects (**C** and **D**, respectively) elicited by intravenous (i.v.) administration of 1-nitro-2-phenylethene (NPe; 3 mg/kg) in four groups of pentobarbital-anesthetized spontaneously hypertensive rats: i) Before and after bivagotomy (Biv) at cervical level, ii) before and after perineural pretreatment (PNT) of both cervical vagi with capsaicin (250 μg/ml), iii) before and after pretreatment with capsazepine (Capz; 1 mg/kg, i.v.), and iv) before and after pretreatment with ondansetron (Ond; 30 μg/kg, i.v.). Data are the mean ± SEM and expressed as a percentage of baseline (*n* = 6 rats per group). *P < 0.05 and ^#^ *P* < 0.05 by paired Student’s *t* test vs. the respective baseline values and the corresponding responses before any pretreatment, respectively.

**Figure 5:**
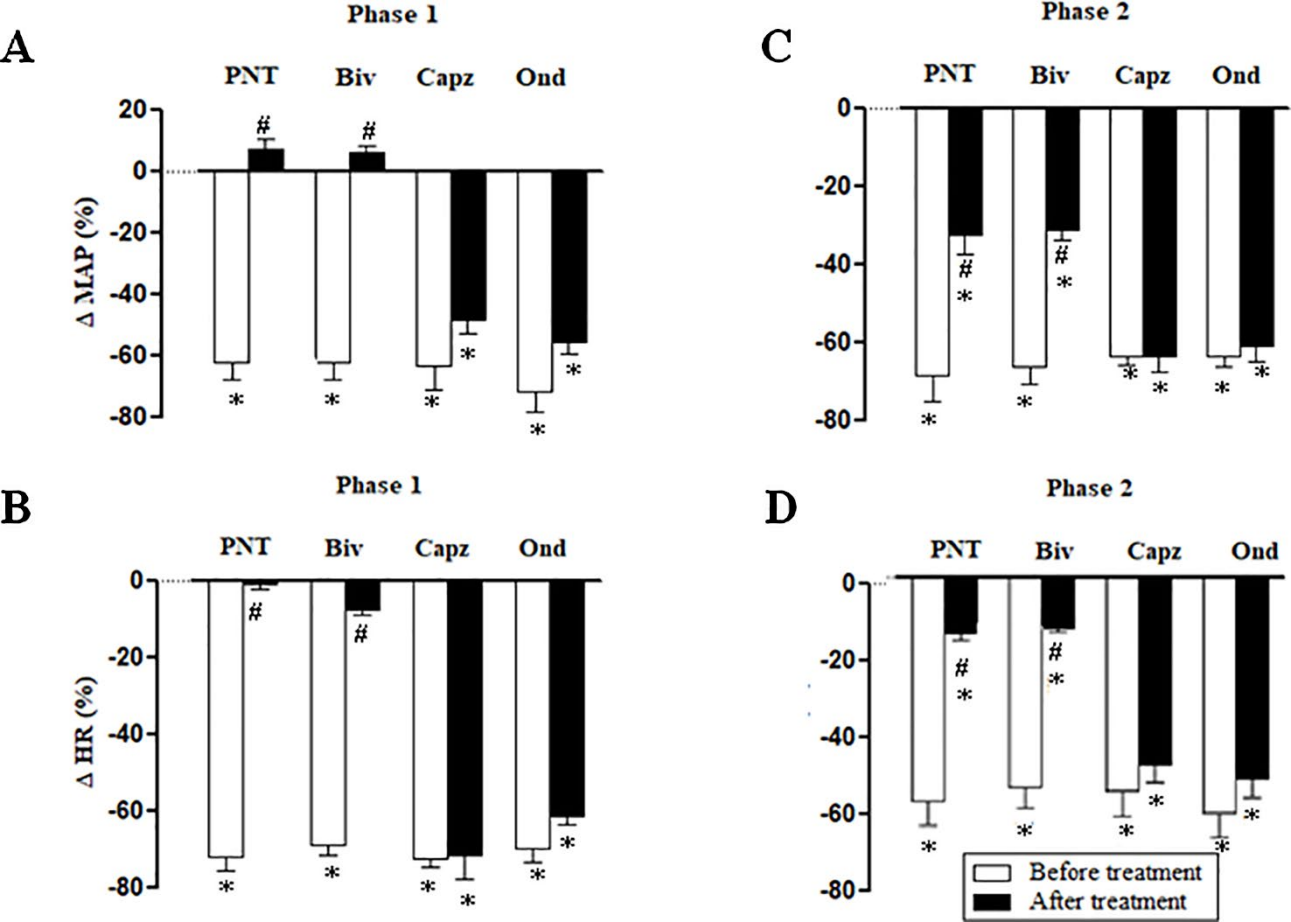
Phase 1 decreases in mean arterial pressure (ΔMAP) and heart rate (ΔHR) (**A** and **B**, respectively) and phase 2 hypotensive and bradycardiac effects (**C** and **D**, respectively) elicited by intravenous (i.v.) administration of *trans*-4-methoxy-β-nitrostyrene (T4MN; 0.3 mg/kg) in four groups of pentobarbital-anesthetized spontaneously hypertensive rats: **i**) before and after bivagotomy (Biv) at cervical level, ii) before and after perineural pretreatment (PNT) of both cervical vagi with capsaicin (250 μg/ml), iii) before and after pretreatment with capsazepine (Capz; 1 mg/kg, i.v.), and iv) before and after pretreatment with ondansetron (Ond; 30 μg/kg, i.v.). Data are the mean ± SEM and expressed as a percentage of baseline (*n* = 6 rats per group). *P < 0.05 and ^#^ *P* < 0.05 by paired Student’s *t* test vs. the respective baseline values and the corresponding responses before any pretreatment, respectively.

Both cervical bivagotomy and PNT with capsaicin fully prevented the phase 1 hypotensive and bradycardiac effects of NPe (**Figures 4A, B**, respectively) and T4MN (**Figures 5A, B**, respectively) and significantly reduced the phase 2 of hypotensive and bradycardiac responses to NPe (**Figures 4C, D**, respectively) and T4MN (**Figures 5C, D**, respectively) (*P* < 0.05, paired Student’s test). In contrast, i.v. pretreatment with capsazepine was inert (*P* > 0.05, paired Student’s test) on both phases 1 and 2 of hypotensive and bradycardiac responses to NPe (**Figure 4**) and T4MN (**Figure 5**). The same was observed for the influence of i.v. pretreatment with ondansetron on phase 1 and 2 cardiovascular responses to either NPe (**Figure 4**) or T4MN (**Figure 5**). It is noteworthy that in positive control experiments, the doses of both capsazepine (1 mg/kg, i.v.) and ondansetron (30 µg/kg, i.v.) used in the present study were able to reduce or even abolish the well-known vago-vagal reflex induced by capsaicin (1 µg/kg, i.v.) and 5-HT_3_ (10 µg/kg, i.v.), respectively (data not shown). Similar findings were previously reported in studies performed in normotensive rats (de Siqueira et al., 2006; de Siqueira et al., 2010; Ribeiro-Filho et al., 2016).

### Cardiovascular Responses to NPe and T4MN Injected Into the Left Ventricle in Anesthetized SHRs (Series 6)

**Figure 6:**
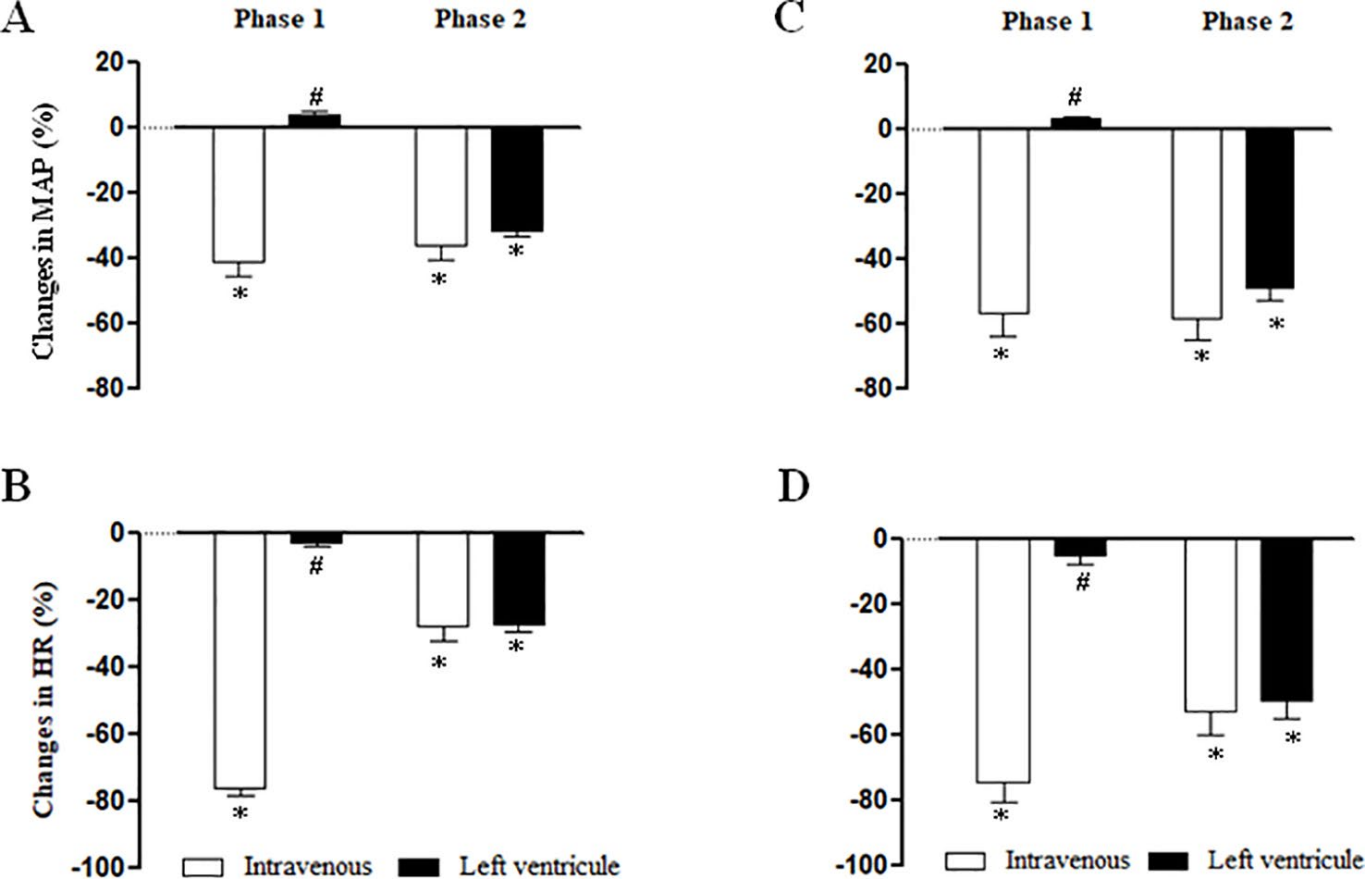
Maximal changes in mean arterial pressure (MAP) and heart rate (HR) induced by 1-nitro-2-phenylethene (NPe, 3 mg/kg) (**A** and **B**, respectively) and *trans*-4-methoxy-β-nitrostyrene (T4MN; 0.3 mg/kg) (**C** and **D**, respectively) injected into the left ventricle in pentobarbital-anesthetized spontaneously hypertensive rats which have been submitted 15 min earlier to an intravenous injection of NPe (3 mg/kg) or T4MN (0.3 mg/kg) (positive control). Data are expressed as the mean ± SEM and expressed as percentage of baseline (*n* = 5 per group). *P < 0.01 by paired Student’s *t* test vs. the corresponding baseline values. ^#^ *P* < 0.001 by Mann–Whitney *U* test vs. the corresponding responses to i.v. administration, respectively.

**Figure 6** summarizes the maximal MAP and HR elicited by NPe (3 mg/kg) (panels **A** and **B**, respectively) and T4MN (0.3 mg/kg) (panels **C** and **D**, respectively) injected into the left ventricle in pentobarbital-anesthetized SHRs, which have been submitted 10 min earlier to an i.v. injection of the same doses of NPe or T4MN. After injection into the left ventricle, NPe and T4MN no longer evoked the phase 1 cardiovascular responses (**Figure 6**). However, following intraventricular injection of NPe and T4MN, a significant hypotension (**Figures 6A, C**, respectively) and bradycardia (**Figures 6B, D**, respectively) were recorded (*P* < 0.01 by paired Student’s test). These responses have the same time of latency and were of the same order of magnitude when compared to the corresponding responses evoked by systemically-injected NPe and T4MN (**Figure 6**).

### Effects of Intravenous Pretreatment With Methylatropine on the Hypotensive and Bradycardiac Responses to NPe and T4MN in Conscious SHRs (Series 7)

**Figure 7:**
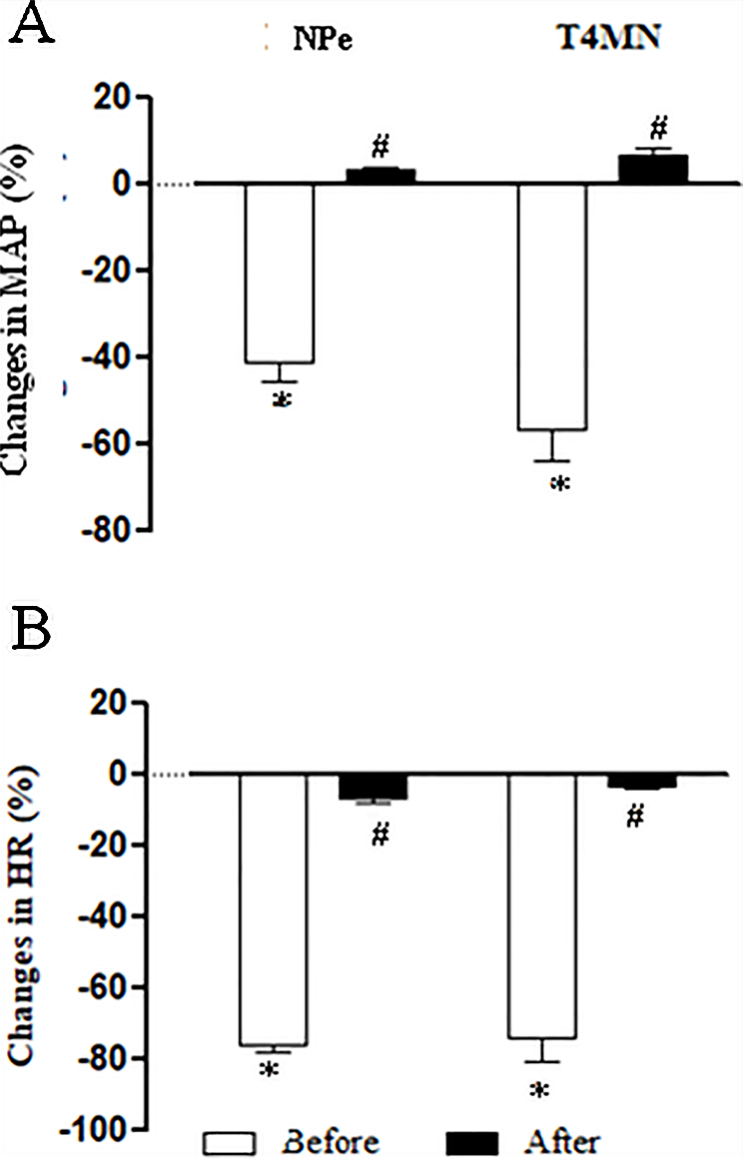
Maximal decreases in mean arterial pressure (MAP) **(A)** and heart rate (HR) **(B)** induced by intravenous (i.v.) injection of 1-nitro-2-phenylethene (NPe; 3 mg/kg) and *trans*-4-methoxy-β-nitrostyrene (T4MN; 0.3 mg/kg) in conscious, freely moving spontaneously hypertensive rats pretreated with i.v. vehicle or methylatropine (1 mg/kg). Data are expressed as the mean ± SEM and expressed as percentage of baseline (*n* = 5). *P < 0.001 by paired Student’s *t* test vs. the corresponding baseline values. ^#^ *P* < 0.001 by Mann–Whitney *U* test vs. corresponding responses before any pretreatment.

The average baseline values of MAP and HR in conscious SHRs before any treatment are shown in **Table 1**. Bolus injection of NPe (3 mg/kg) and T4MN (0.3 mg/kg), but not their vehicle (data not shown), evoked significant (*P* < 0.01, paired Student’s test) monophasic hypotension (**Figure 7A**) and bradycardia (**Figure 7B**). As was observed in anesthetized SHRs, the time latency of these effects was about 1–2 s after the injection of both nitroderivatives. As expected, only baseline HR values were significantly enhanced following i.v. pretreatment of conscious SHRs with 1 mg/kg methylatropine (*P* < 0.05, paired Student’s test; **Table 1**). In these animals, phase 1 hypotension and bradycardia (**Figures 7A, B**, respectively) was not recorded following i.v. injection of either NPe or T4MN.

## Discussion

In the present study, baseline MAP and HR recorded in both conscious and pentobarbital-anesthetized SHRs were similar to those previously reported (Interaminense et al., 2011; de Barros Correia Junior et al., 2015). Magnitude of cardiovascular responses to i.v. NPe and T4MN in conscious SHRs was of the same order of magnitude as those recorded in anesthetized ones. This suggests that, unlike other anesthetic agents, pentobarbital anesthesia is preferable to assess cardiac function in rats since it induced less influence on baseline HR, as previously reported (Kawahara et al., 2005; Stein et al., 2007; Sano et al., 2016). As previously reported for NPa (de Siqueira et al., 2010; Interaminense et al., 2011), i.v. injection of NPe (doses of ≥0.3 mg/kg) and T4MN (doses of ≥0.05 mg/kg) elicited biphasic hypotension and bradycardiac effects. Essentially, a fast component (phase 1) followed by a slow component (phase 2) with a time of latency of 1–2 s and nearly 7–9 s, respectively. To the best of our knowledge, this is the first time that the reversible cardiovascular effects of NPe and T4MN are reported in SHRs.

The present study focused on the immediate and rapid bradycardia that occurred concomitantly with the initial hypotension (phase 1). We found that this phase 1 cardiovascular responses to NPe and T4MN occurred with a time latency similar to that of the vago-vagal reflex to i.v. capsaicin (Donnerer and Lembeck, 1982; Yang et al., 1993) or serotonin (Owen et al., 2005), leading us to suggest that it could be also from a reflex origin. This hypothesis was strengthened by the observation that the phase 1 decreases in HR occurred before the concomitant hypotension (1 vs. 2 s, respectively). Indeed, since the selective blockade of neural conduction of vagal afferent by PNT with capsaicin abolished the phase 1 cardiovascular responses as did the cervical bivagotomy, it is presumed that the afferent limb of this reflex is vagal. On the other hand, the phase 1 hypotension and bradycardia evoked by both NPe and T4MN was sensitive to the peripheral muscarinic receptor antagonist methylatropine, indicating that it is mediated by acetylcholine released from the endings of vagal efferent nerves. Taken together, these results demonstrate that phase 1 hypotension and bradycardiac reflex is mediated through a vago-vagal pathway, as previously reported for other nitroderivatives such NPa (de Siqueira et al., 2010; Interaminense et al., 2011) and *trans*-methyl-β-nitrostyrene (Teófilo et al., 2019).

Unlike in anesthetized SHRs, i.v.-administrated NPe and T4MN in conscious SHRs induced a brief monophasic hypotension and bradycardia. Such a difference could be explained by the fact that anesthesia with pentobarbital induces decreases in baroreceptor reflex activity (Bedran‐de‐Castro et al., 1990; Barringer and Buñag, 1990). In conscious SHRs, phase 2 hypotensive response to NPe and T4MN may be masked by the increased sensitivity of the baroreflex control. On the other hand, it seems unlikely that activation of vagal sensory C‐fibers by either NPe or T4MN could be mediated indirectly *via* their metabolites and/or other released substances since the time latency of the rapid phase 1 responses evoked by these nitroderivatives is too short for such phenomenon.

We have examined whether the vago-vagal reflex evoked by NPe and T4MN involves activation of sensory pulmonary or cardiac C-fiber afferents. For this purpose, we compared the cardiovascular responses to an intraventricular injection of the nitroderivatives into the left ventricle to those evoked by their systemic injection. We found that, unlike the i.v. route, injection of NPe and T4MN into the left ventricle no longer evoked the rapid hypotension and bradycardia (phase 1). We concluded, therefore, that both nitroderivatives evoked the phase 1 vago-vagal reflex through stimulation of vagal afferent nerves to the lungs since their intraventricular injection into the left ventricle bypasses the pulmonary circulation with no immediate access to the pulmonary C-fibers. Activation of vagal pulmonary afferents by different types of chemical stimulants is known to induce pronounced respiratory and cardiovascular reflex responses in rats (Dawes and Comroe, 1954; Coleridge and Coleridge, 1984; Brugere et al., 1986).

It is well known that i.v. injection of the selective TRPV_1_ receptor agonist capsaicin (Donnerer and Lembeck, 1982; Yeh et al., 1993) or the 5‐HT_3_ receptor agonist serotonin (Whalen et al., 2000) induced immediate and rapid hypotension and bradycardia (phase 1) in rats. In order to assess the receptor specificity of the vago-vagal reflex induced by NPe and T4MN, experiments were conducted in anesthetized SHRs which have been pretreated intravenously with the TRPV_1_ receptor antagonist capsazepine (Bevan et al., 1992; Malinowska et al., 2001) and the 5-HT_3_ receptor antagonist ondansetron (Bagchi and Deshpande, 1999). Our results showed that both NPe- and T4MN-induced vago-vagal reflex did not involve the activation of vanilloid TRPV_1_ and 5‐HT_3_ receptors (and/or secondary to release of endogenous 5‐HT) since it remained unchanged following pretreatment with capsazepine and ondansetron, respectively. These results are consistent with previous reports conducted by us with NPa in either normotensive (de Siqueira et al., 2010) or SHRs (Interaminense et al., 2011). At the present time, the receptors expressed on the sensory vagal afferents responsible for NPe- and T4MN-induced vago-vagal cardiovascular reflex are unknown. It was reported that activation of ankyrin subtype 1 (TRPA_1_) receptors with i.v. cinnamaldehyde evoked a transitory hypotensive and bradycardiac effects (Bezold–Jarisch-like reflex) in anesthetized mice (Pozsgai et al., 2010). Furthermore, i.v.-administered α,β-methyleneadenosine 5′-triphosphate (McQueen et al., 1998) induced reflex bradycardia and hypotension in anesthetized rats. Further experiments are necessary to address whether putative stimulation of TRPA_1_ or purinergic P2X receptors is involved in mediation of phase 1 hypotensive and bradycardiac effects of NPe and T4MN in SHRs.

As previously suggested for NPa (de Siqueira et al., 2010), several lines of evidence support the hypothesis that phase 2 hypotension induced by NPe and T4MN is partially due to their direct vasodilatory action upon the vascular smooth muscle. First, phase 2 hypotensive response to i.v.-administered NPe and T4MN is most likely due to the decrease in the peripheral vascular resistances since it was more potent on diastolic arterial blood pressure (**Figure 3**). Second, a significant decrease in MAP was recorded when NPe and T4MN were injected into the left ventricle. This hypotension has the same time of latency and was of the same order of magnitude as the phase 2 hypotension elicited by systemically-injected NPe and T4MN. Finally, *in vitro* experiments showed that NPe (Alves-Santos, 2017) and T4MN (Alves-Santos et al., 2019) elicited concentration‐dependent vasorelaxation in small resistance arteries from SHRs. Vasorelaxant effects of T4MN in small resistance arteries from SHRs are mediated through both endothelium-dependent (activation of Akt/eNOS/NO pathway) and endothelium-independent (activation of sGC/cGMP/PKG pathway) mechanisms (Alves-Santos et al., 2019). A similar mechanism was advanced for the vasorelaxant activity of NPe in the same preparation from SHRs (Alves-Santos, 2017).

In the present investigation, the vago-vagal reflex elicited by NPe was recorded only from a dose of 0.3 mg/kg, while that evoked by T4MN was evident from a dose of 0.05 mg/kg (**Figure 2**). Indeed, we found no statistical difference when the magnitude of this reflex induced by 3 mg/kg NPe was compared to that evoked by 0.1 mg/kg T4MN (**Figure 2**). Thus, with the presence of an electron-releasing group bonded in its aromatic ring, T4MN appears about 30 times more potent for the recruitment of chemosensitive pulmonary vagal afferent fibers than its parent drug NPe. A similar profile was recently reported in isolated small resistance arteries from SHRs since we found that T4MN was 22 times more potent as a relaxing agent than NPe (Alves-Santos et al., 2019). The present results further our knowledge about the cardiovascular effects of T4MN and may be relevant to the characterization of promising new nitro compounds for the treatment of cardiovascular diseases.

It is concluded that i.v.-administered NPe and T4MN elicited a vago-vagal hypotensive and bradycardic reflex (phase 1) that did not involve the activation of vanilloid TRPV_1_ or 5-HT_3_ receptors located on vagal pulmonary sensory nerves. T4MN was more potent in inducing this reflex, a finding that seems indicative that insertion of an electron donor such as a methoxy group into the aromatic ring stabilized the NPe molecule, which in turn increases its hypotensive and bradycardiac effects. Phase 2 hypotensive response to i.v. NPe and T4MN seems partially resulting from a direct vasodilatory action.

## Data Availability Statement

All datasets generated for this study are included in the article/supplementary material.

## Ethics Statement

The animal study was reviewed and approved by the institutional animal ethics committee (23076.050472/2012-77).

## Author Contributions

TA-S: Performed all experiments in anesthetized and conscious rats, responsible for the acquisition, analysis and interpretation of data. Participated in the redaction and revision of the article. OS: Participated in experiments in conscious rats, responsible for the acquisition, analysis and interpretation of the data and approval of final version. HM: Participated in experiment with intraventricular experiments, responsible for the acquisition, analysis and interpretation of the data and approval of final version. RB: Performed synthesis of the nitroderivatives, contributed with new reagents and analytical tools, and participated in the revision and approval of final version. GD, PM, SL: Supervision, analysis and interpretation of data, responsible for the concept and design of the manuscript, and for the preparation of the manuscript. Participated in the redaction and revision of the article.

## Funding

This work was supported by the “Conselho Nacional de Pesquisa (CNPq, Edital Universal 14/2012, grant number 484656/2012-0),” the “Coordenação de Aperfeiçoamento de Pessoal de Nível Superior-Brasil (CAPES; Finance Code 001), and the “Fundação Amazônica de Amparo a Estudos e Pesquisas” (FAPESPA, ICAAF, grant number 177/2014). This study is part of a Doctoral Thesis developed by TA-S in order to obtain his Ph.D. degree in Biochemistry and Physiology at the Federal University of Pernambuco, Recife, Brazil. Expert reviewing of the manuscript by Dr. Steven D. Aird is gratefully acknowledged.

## Conflict of Interest

The authors declare that the research was conducted in the absence of any commercial or financial relationships that could be construed as a potential conflict of interest.
